# Coal mining environment causes adverse effects on workers

**DOI:** 10.3389/fpubh.2024.1368557

**Published:** 2024-04-29

**Authors:** Huihui Chen, Xinping Ding, Wenzhong Zhang, Xichen Dong

**Affiliations:** ^1^Wannan Medical College, Wuhu, Anhui, China; ^2^Guang’anmen Hospital China Academy of Chinese Medical Sciences, Beijing, China; ^3^Huaibei Occupational Disease Prevention and Control Institute, Huaibei, Anhui, China

**Keywords:** coal mining environment, immunity, renal function, apoptosis, anemia

## Abstract

**Background:**

The objective of this study is to study the adverse effects of coal mining environment on workers to discover early effective biomarkers.

**Methods:**

The molecular epidemiological study was conducted with 502 in-service workers, who were divided into miner and auxiliary. We measured the individual levels of dust exposure for participants. Clinical examinations were conducted by qualified doctors. Peripheral blood was collected to measure biochemistry, hemogram, and karyocyte apoptosis.

**Results:**

All workers were healthy who have not found with any diseases that can be diagnosed medically in the physical examination and showed no difference in dust exposure level, age, height, weight, and body mass index between groups. The working years of miners were lower than that of auxiliaries (*p* < 0.001). Compared with auxiliaries, the concentration and percentage of lymphocytes (*p* = 0.040, *p* = 0.012), basophils (*p* = 0.027, *p* = 0.034), and red blood cells (*p* < 0.001) and the concentration of hemoglobin of miners were lower (*p* < 0.001). The percentage of neutrophils (*p* = 0.003), the concentration of mean corpuscular hemoglobin concentration (*p* = 0.002), and the proportion of karyocyte apoptosis in miners were higher (*p <* 0.001). Miners presented higher blood urea nitrogen (*p* < 0.001), ratio of blood urea nitrogen to creatinine (*p* < 0.001), the high density lipoprotein cholesterol (*p* < 0.001), lower creatinine (*p* < 0.05), and cholesterol (*p* < 0.001).

**Conclusion:**

The coal mining environment impacted mining workers’ immune function, renal function, and the hematopoietic system, including BUN/CRE, HGB, RBC, and LYMPH, which could be used as early biomarkers to screen the health of coal miners.

## Introduction

In coal mining activities, a range of occupational hazards including coal mine dust, exhaust gas, high temperature, high humidity, and noise form the coal mining environment (1). Respirable dust refers to dust particles with an aerodynamic diameter of less than 5 μm that can reach the deep respiratory tract and alveolar region, and the concentration of respirable dust directly affects the health of workers. Coal mine dusts are supervised by the government, and their concentrations are controlled under maximum limit standard in order to protect the health of workers. However, coal mine dust exposure leads to pneumoconiosis, posing the primary threat to miners’ health (2). Additionally, coal mine dust contains a variety of heavy metals that can contaminate the environment during coal mining and transportation processes (3). Heavy metals accumulate in multiple organs and tissues of humans and animals (4). Among them, the kidney is the main target organ for heavy metal accumulation (4). Batool et al. (5) report that coal mine dust can cause inflammation and oxidative damage to miners. Except heavy metal elements, exhaust gases cause oxidative damage (6). Oxidative damage results in DNA damage, which is a significant factor for the onset of cancer and chronic diseases (7). Therefore, the combination effects of multiple hazards in the coal mining environment may lead to yet unnoticed health implications. To date, the scientific community has yet to identify specific biomarkers for the screening of sub-health conditions among coal miners within the coal mining environment. This gap underscores the critical need for a deeper understanding of the health impacts of coal mining operations and enhancements in health monitoring methodologies.

Consequently, the aim of this study is to comprehensively examine the influence of the coal mining environment on the health biomarkers of miners, with the goal of identifying particular biomarkers that could serve as early indicators of health issues.

## Methods

### Cross-sectional study

This study was approved by the Ethics Committee of Huaibei Occupational Disease Prevention and Control Institute (approval number: 20180603), and written informed consent was obtained from each participant. The study confirmed that all experiments were conducted in accordance with relevant guidelines and regulations. In 2018, a molecular epidemiological study was conducted on 502 in-service workers recruited from Huaibei Mining Group in Anhui Province, China. Face-to-face survey was conducted by trained investigators using a unified questionnaire. The general information including eating habits, history of smoking, drinking, family, and occupation was collected. In this study, 279 people directly contacted with the coal mine were considered as miners including tunneling workers, drilling workers, and winch drivers, while 223 people indirectly contacted with the coal mine were considered as auxiliaries including office workers, loading workers, and warehouse keepers. All workers use the unified personal protective equipment provided by the company in workplace. The occupational hazards of coal mining environment including respirable dust were monitored. According to the “Specifications of air sampling for hazardous substances monitoring in the workplace (GBZ 159–2004)” and the “Method for determination of dust in the air of workplace. Part 2: respirable dust concentration (GBZ / T 192.2–2007)”, the permissible concentration-time weighted average was measured for 23 miners and 28 auxiliaries based on individual samplers of dust, after continuously collecting for three working days in coal mines (8, 9).

### Clinical examination

Physical examinations (including medical examinations, surgical examinations, and respiratory functions), imaging examinations (including electrocardiogram, chest X-ray, and abdominal ultrasound), and blood collection were carried out by qualified doctors in Huaibei Occupational Disease Prevention and Control Institute.

### Hematology and blood biochemistry

After participants’ overnight fasting, 5 mL of peripheral venous blood was collected to determine biomarkers. Hematological parameters of whole blood were measured with an automatic hemocytometer (Nihon Kohden MEK-6813 K). Blood biochemistry parameters were analyzed with an automated biochemical analyzer (AU680, Beckman Coulter Ltd.).

### Apoptosis or necrosis analysis by alkaline comet assay

We used CometA.1 comet image intelligent analysis software (Beijing Huaxing Innovation Technology Co., Ltd.) to analyze the cell images. Comets were visualized using a light microscope with 510 nm excitation and 590 nm emission filters. The software can analyze the results by indicators, such as total comet length, head length, tail length, tail DNA percentage, and tail length/head length ratio. We used the tail DNA percentage indicator as the judgment indicator in this experiment. Each sample was analyzed for 150 cells, and the number of “spiky” cells observed in the field of view was recorded. Cells with a tail DNA percentage of more than 95% were regarded as apoptosis, and then, the apoptosis rate was determined.

### Statistical analysis

Homogeneity of variances was analyzed by Levene’s test. Because most variances of miners and auxiliaries were non-normal distribution, group differences were conducted with Wilcoxon test; data were expressed with quantile, including median, q25 and q75. The Spearman correlation was carried out between work type and parameters, as well as karyocyte apoptosis and lymphocytes (LYMPH). All reported *p*-values were two-tailed, and *p*-value of less than 0.05 was considered as statistically significant.

## Results

### Exposure measurement

All workers’ time weighted average concentrations were within occupational exposure limits, and there was no obvious difference between miners and auxiliaries (Table 1).

**Table 1:** Time weighted average concentrations of respirable dust.

Work type	No.	Qualified samples	Pass ratio (%)	Results (mg/m^3^)		
				Minimum	Maximum	Median
Miner	23	23	100	0.96	2.49	1.68
Auxiliary	28	28	100	0.40	2.40	1.61

### General information

All workers were male, passed normal clinical physical examinations, and found no signs of diseases related to coal mine dust in the medical examination, and there were no differences in age, systolic pressure, pulse frequency, height, weight, and body mass index between miners and auxiliaries (*p* > 0.05). The working years of miners were significantly lower than that of auxiliaries (*p* < 0.001). Miners’ diastolic pressure was lower, and pulse pressure difference was higher than that of auxiliaries; all parameters were in normal range (Table 2).

**Table 2:** General information results of miners and auxiliaries presented as quartile (median, q25, q75).

Parameters	Auxiliary	Miner	*p*-value
Age (year)	45.0 (35.0; 50.0)	44.0 (39.0; 48.0)	0.934
Length of work (year)	11.0 (8.00; 21.0)	9.00 (3.25; 13.0)	0.000**
Body mass index (kg/m^2^)	24.3 (22.5; 26.2)	23.8 (22.0; 26.0)	0.071
Systolic pressure (mmHg)	129.0 (119.5; 139.0)	129.0 (120.0; 139.0)	0.808
Diastolic pressure (mmHg)	80.0 (74.0; 88.0)	78.0 (73.0; 86.0)	0.038*
Pulse pressure difference (mmHg)	48.0 (40.8; 55.0)	50.0 (45.0; 57.0)	0.000**
Pulse frequency (times/min)	78.0 (71.0; 86.0)	77.0 (69.0; 85.0)	0.075
Height (cm)	170.0 (168.0; 175.0)	170.0 (168.0; 175.0)	0.756
Weight (kg)	71.0 (65.0; 78.0)	70.0 (64.0; 76.0)	0.096

*Compared with auxiliary *p* < 0.05, **Compared with auxiliary *p* < 0.001.

### Hemogram

Compared with auxiliaries, the concentration and percentage of LYMPH (*p* = 0.040, *p* = 0.012), basophils (BASO) (*p* = 0.027, *p* = 0.034), and red blood cells (RBC) (*p* < 0.001) and the concentration of hemoglobin (HGB) (*p* < 0.001) of miners were lower. The percentage of neutrophils (NEUT) (*p* = 0.003) and the concentration of mean corpuscular hemoglobin concentration (MCHC) (*p* = 0.002) were significantly higher. Parameters of white blood cells (WBC), mean corpuscular volume (MCV), platelet (PLT), mean platelet volume (MPV), platelet hematocrit (PCT), mean corpuscular hemoglobin (MCH), concentration of neutrophils (NEUT), and concentration and percentage of monocyte macrophage (MONO) and eosinophils (EO) presented no difference between miners and auxiliaries (*p* > 0.05) (Table 3).

**Table 3:** Hemogram results of miners and auxiliaries presented as quartile (median, q25, q75).

Parameters	Auxiliary	Miner	p-Value
WBC (10^9^/L)	6.37 (5.31; 7.55)	6.20 (5.30; 7.37)	0.604
RBC (10^12^/L)	5.03 (4.78; 5.27)	4.85 (4.59; 5.10)	0.000**
HGB (g/L)	153.5 (147.0; 161.0)	149.0 (142.0; 156.0)	0.000**
MCV (fL)	94.5 (91.8; 97.2)	94.1 (91.4; 96.2)	0.095
MCHC (g/L)	325.0 (319.3; 330.0)	327.0 (322.0; 333.0)	0.002*
PLT (10^12^/L)	242.5 (209.8; 278.5)	242.0 (206.0; 272.0)	0.654
MPV (fL)	10.6 (10.0; 11.2)	10.6 (10.0; 11.3)	0.761
PCT (%)	0.26 (0.23; 0.29)	0.26 (0.23; 0.28)	0.566
MCH (pg)	30.8 (29.8; 31.6)	30.7 (29.8; 31.7)	0.946
NEUT (10^9^/L)	3.61 (2.87; 4.38)	3.68 (2.74; 4.70)	0.456
NEUT (%)	55.4 (49.9; 60.2)	57.3 (51.9; 62.2)	0.003*
LYMPH (10^9^/L)	2.11 (1.76; 2.53)	2.01 (1.70; 2.35)	0.040*
LYMPH (%)	34.0 (29.3; 38.8)	32.1 (28.0; 37.1)	0.012*
MONO (10^9^/L)	0.46 (0.37; 0.55)	0.46 (0.38; 0.56)	0.641
MONO (%)	7.10 (6.20; 8.25)	7.30 (6.25; 8.40)	0.184
EO (10^9^/L)	0.13 (0.07; 0.20)	0.10 (0.06; 0.18)	0.068
EO (%)	2.00 (1.20; 3.00)	1.75 (0.93; 2.70)	0.060
BASO (10^9^/L)	0.03 (0.02; 0.05)	0.03 (0.02; 0.04)	0.027*
BASO (%)	0.50 (0.40; 0.70)	0.50 (0.30; 0.70)	0.034*

*Compared with auxiliary *p* < 0.05, **Compared with auxiliary *p* < 0.001.WBC, White Blood Cells; RBC, Red Blood Cells; HGB, Hemoglobin; MCV, Mean Corpuscular Volume; MCHC, Mean Corpuscular Hemoglobin Concentration; PLT, Platelet. MPV, Mean Platelet Volume. PCT, Platelet Hematocrit. MCH, Mean Corpuscular Hemoglobin. NEUT, Neutrophils. LYMPH, Lymphocytes. MONO, Monocyte Macrophage. EO, Eosinophils; BASO, Basophils.

### Blood biochemistry parameters

**Figure 1:**
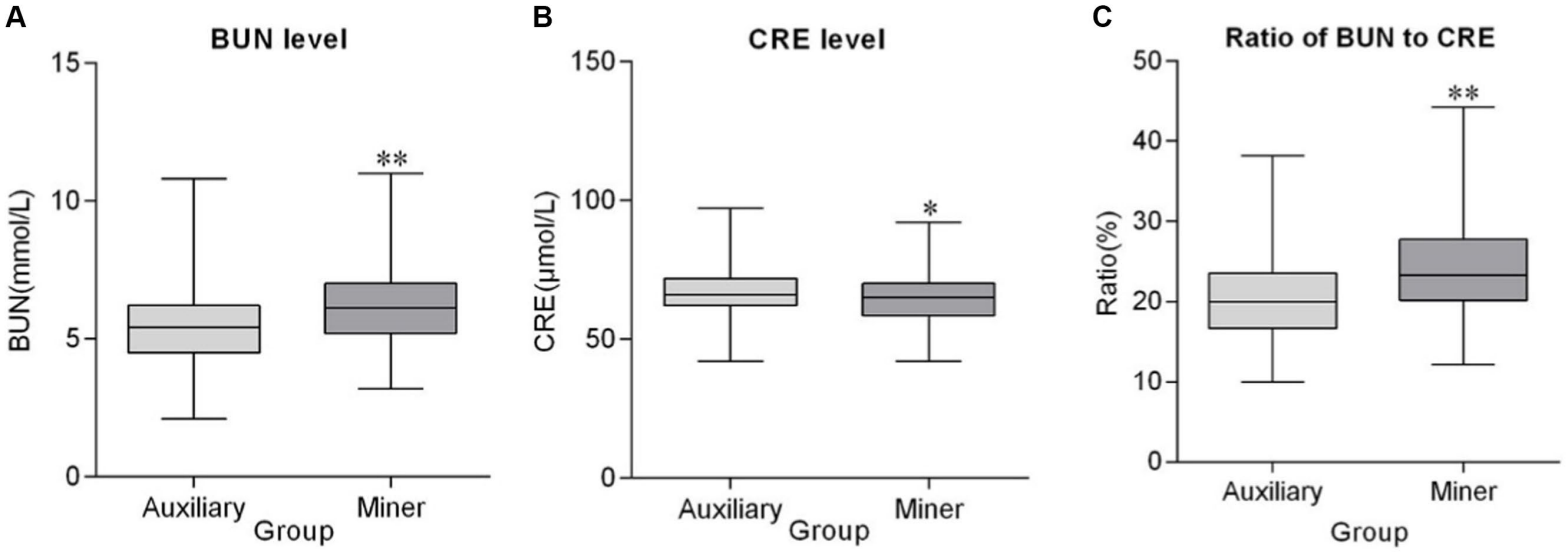
Renal function indexes of miners and auxiliaries. *Compared with auxiliary *p* < 0.05, **Compared with auxiliary *p* < 0.001.

Compared with auxiliaries, blood urea nitrogen (BUN) (*p* < 0.001) and ratio of blood urea nitrogen to creatinine (BUN/CRE) (*p* < 0.001) were higher, and creatinine (CRE) (*p* < 0.05) was lower in miners (Figure 1). The parameters of alanine aminotransferase (ALT) (*p* = 0.010) and cholesterol (CHO) (*p* < 0.001) of miners were lower. Moreover, high density lipoprotein cholesterol (HDL-C) (*p* < 0.001) was significantly higher. The parameters of aspartate aminotransferase (AST), albumin (ALB), total bilirubin (TBIL), alkaline phosphatase (ALP), fasting blood glucose (GLU), triglyceride (TG), and low density lipoprotein cholesterol (LDL-C) presented no difference between miners and auxiliaries (*p* > 0.05). Additionally, lower ALT was regarded as no biological meaning (Table 4).

**Table 4:** Blood biochemistry indicator results of miners and auxiliaries presented as quartile (median, q25, q75).

Parameters	Auxiliary	Miner	*p*-value
ALT (U/L)	25.0 (18.0; 33.0)	22.5 (16.0; 29.0)	0.010*
AST (U/L)	22.0 (18.3; 26.0)	22.0 (19.0; 26.0)	0.608
ALB (g/L)	46.0 (44.0; 47.0)	46.0 (44.0; 47.0)	0.122
TBIL (umol/L)	11.6 (8.80; 15.8)	10.7 (8.50; 14.7)	0.082
ALP (U/L)	84.0 (72.0; 100.0)	84.0 (75.0; 96.0)	0.946
GLU (mmol/L)	5.10 (4.80; 5.40)	5.00 (4.70; 5.40)	0.349
CHO (mmol/L)	4.30 (3.29; 5.11)	3.80 (1.40; 4.72)	0.000**
TG (mmol/L)	1.26 (0.82; 1.74)	1.30 (0.85; 1.75)	0.904
HDL-C (mmol/L)	1.38 (1.19; 1.76)	1.59 (1.31; 2.12)	0.000**
LDL-C (mmol/L)	2.61 (2.10; 3.07)	2.51 (2.13; 2.87)	0.213

*Compared with auxiliary *p* < 0.05, **Compared with auxiliary *p* < 0.001.ALT, Alanine Aminotransferase; AST, Aspartate Aminotransferase; ALB, Albumin; TBIL, Total Bilirubin, ALP, Alkaline Phosphatase; GLU, Glucose; CHO, Cholesterol; TG, Triglyceride; HDL-C, High Density Lipoprotein Cholesterol. LDL-C, Low Density Lipoprotein Cholesterol.

### Karyocyte apoptosis in peripheral blood

**Figure 2:**
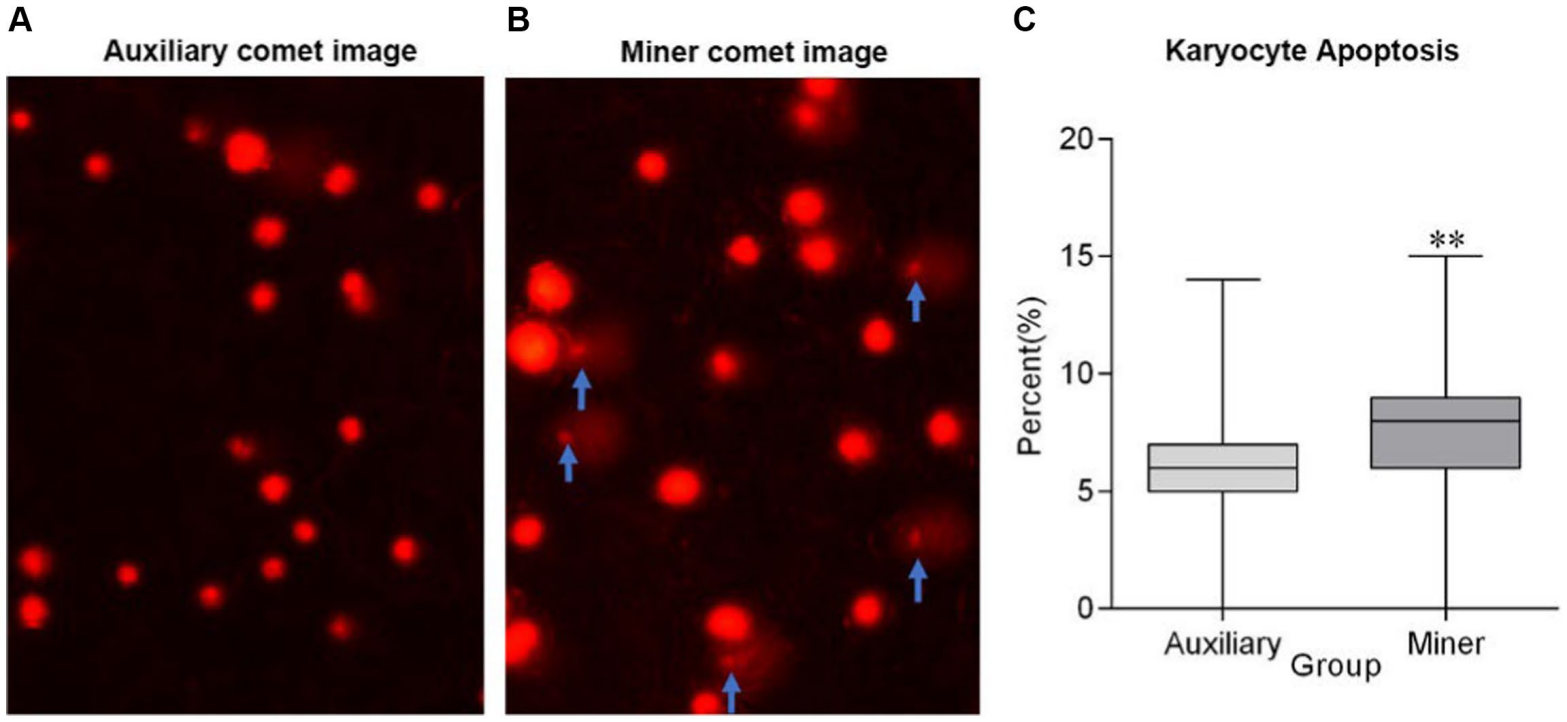
Apoptotic cells in peripheral blood of miners and auxiliaries. *Compared with auxiliary *p* < 0.05, **Compared with auxiliary *p* < 0.001, the apoptotic cells marked with the arrow.

Compared with auxiliaries, the percentage of apoptotic cells in peripheral blood was significantly higher in miners (*p* < 0.001) (Figure 2).

### Correlation analysis between work type and parameters

The correlation with work type was sequenced with karyocyte apoptosis, BUN/CRE, HGB, RBC, and LYMPH (Table 5).

**Table 5:** Correlation between work type and parameters.

Correlation	Apoptosis cell ratio	Ratio BUN/ CRE	Hemoglobin	RBC	Absolute value of lymphocytes
*r*_s_	0.385	0.315	−0.224	−0.216	−0.092
Sig.	0.000**	0.000**	0.000**	0.000**	0.040*

*r*_s_ means correlation coefficient, *Compared with auxiliary *p* < 0.05, **Compared with auxiliary *p* < 0.001.

## Discussion

With the increasing mechanization level in coal mining, the pollution from exhaust gas has become more severe. Exhaust gases are harmful to health; however, lack of relevant regulation is difficult to monitor the exhaust gas (6). In the present study, coal mine dust is within occupational exposure limits in coal mining environment.

A 10-year cohort study was conducted in Henan province of China, with 12,000 male miners, showed that the risk of hypertension in miners was related to the level of coal mine dust exposure (10). Additionally, epidemiological study has found that the prevalence of cardiovascular diseases in miners is higher than other workers (11). In addition, studies have shown that the poor living habits and stressful working environment of miners can easily lead to dyslipidemia (12, 13). Additionally, excessive caloric intake and insufficient exercise result in abnormal blood lipid metabolism (14). The present study showed no cardiovascular diseases in miners; diastolic pressure of miners was lower than that of auxiliaries. The increase in pulse pressure difference among miners suggests that the coal mining environment may adversely affect blood vessels (15). In the present study, lower CHO and higher HDL-C in miners suggest that the coal mining environment does not induce adverse effects in cardiovascular system and lipid metabolism.

Bhuiyan et al. (16) reported that water in proximity to coal mines is contaminated with heavy metals, such as copper, lead, chromium, and cadmium. These pollutants primarily accumulate in the kidneys of fish residing in these contaminated waters (4). Further research supports these findings, revealing an elevated risk of kidney disease among individuals living in coal mining regions (17). Additionally, studies find that exposure to silica dust increases the risk of chronic kidney disease, and there is a direct relationship between air pollution and kidney disease (18). In the present study, both miners and auxiliary staff, residing in dormitories or apartments within the same community close to the coal mine, are subjected to have similar environmental influences. BUN and BUN/CRE are biomarkers for renal function, and their elevation is associated with renal impairment (19, 20). The study found that miners had higher BUN and BUN/CRE levels, indicating adverse effects on renal function due to the coal mining environment. In addition to affecting renal health, air pollution is also linked to a higher incidence of anemia, which is characterized by a decrease in RBC and HGB (21, 22). RBC and HGB are biomarkers for anemia, and the present study shows lower RBC and HGB of miners, which indicates that the coal mining environment may enhance the risk of anemia. However, MCHC is different in RBC and HGB, so the change is regarded as no biological meaning.

As a key indicator of immune function, LYMPH reduction means immune functional impairment (23). In addition, EO and BASO are involved in innate and adaptive immune regulation, contributing to homeostasis of the body (24, 25). In the present study, reduction of LYMPH and BASO in miners suggests that the coal mining environment may impair immune system. Given that compromised immunity heightens susceptibility to infections, epidemiological studies and animal experiments have demonstrated that coal mine dust can trigger inflammatory responses in the lungs and cardiovascular system (26, 27). Except for coal mine dusts, exhausted gas also induces inflammatory response (28). NEUT, as the primary immune cells, increase significantly in inflammatory status (29). In this study, the elevated NEUT levels in miners indicate that the coal mining environment prompts a systemic inflammatory response.

Oxidative stress, induced by coal mine dust, exhibits a time-dependent increase among miners and has the potential to cause DNA damage (5, 30). In addition to oxidative stress, DNA damage can also be induced by water-soluble heavy metals including copper, lead, chromium, and cadmium (31, 32). Research indicates that DNA damage is a precursor to karyocyte (cell nucleus) apoptosis (33, 34). In addition to DNA damage, many factors including inflammatory cytokines, coal mine dusts, and high temperature also can induce apoptosis (35–37). Furthermore, karyocyte apoptosis results in the reduction of LYMPH of the population (35). This present study reveals an increase in apoptotic cells and a decrease in LYMPH levels in the peripheral blood of miners, with a strong correlation observed between karyocyte apoptosis and LYMPH reduction. This suggests that karyocyte apoptosis may lead to a decrease in LYMPH, highlighting the adverse effects of the coal mining environment on immune function.

## Conclusion

Our study reveals five putative occupational biomarkers that correlate with the coal mining environment, namely, karyocyte apoptosis, BUN/CRE, HGB, RBC, and LYMPH, which could be used as early biomarkers to screen for environmental health effects in miners. Thus, the coal mining environment can impact the immune system and renal function and enhance the risk of anemia in miners, which could have significant long-term effects on human health.

### Limitations

Due to the retrospective cohort design utilized in this study, the inability to directly measure all potential risk factors may have compromised the rigor of the outcomes of the study. Therefore, future research will aim to expand the sample size and enhance the monitoring of environmental factors. These adjustments are intended to improve the rigor of the research methodology and the credibility of the findings.

## Data availability statement

The raw data supporting the conclusions of this article will be made available by the authors, without undue reservation.

## Ethics statement

The studies involving humans were approved by the Ethics Committee of Huaibei Occupational Disease Prevention and Control Institute (approval number: 20180603). The studies were conducted in accordance with the local legislation and institutional requirements. The human samples used in this study were acquired from primarily isolated as part of your previous study for which ethical approval was obtained. Written informed consent for participation was not required from the participants or the participants’ legal guardians/next of kin in accordance with the national legislation and institutional requirements. Written informed consent was obtained from the individual(s) for the publication of any potentially identifiable images or data included in this article.

## Author contributions

HC: Writing – original draft, Writing – review & editing, Data curation. XDi: Writing – review & editing. WZ: Supervision, Writing – original draft, Writing – review & editing, Data curation, Investigation, Funding acquisition. XDo: Writing – review & editing, Data curation, Investigation, Supervision, Writing – original draft.
